# Cost and carbon burden of long-acting injections: a sustainable evaluation

**DOI:** 10.1192/pb.bp.114.049080

**Published:** 2016-06

**Authors:** Daniel L. Maughan, Rob Lillywhite, Matthew Cooke

**Affiliations:** 1 Oxford Health NHS Foundation Trust; 2 University of Warwick; 3 Heart of England NHS Foundation Trust

## Abstract

**Aims and method** This study explores the economic cost and carbon footprint associated with current patterns of prescribing long-term flupentixol decanoate long-acting injections. We conducted an analysis of prescription data from a mental health trust followed by economic and carbon cost projections using local and national data.

**Results** A reduction of £300 000 could be achieved across England by improving prescribing behaviour, which equates to £250 per patient per year and 170 000 kg CO_2_e. These savings are unlikely to be released as cash from the service, but will lead to higher-value service provision at the same or lower cost. Most of these carbon emissions are attributable to the carbon footprint of the appointment – 88 000 kg CO_2_e (including energy use and materials used) and the overprescribing of medication – 66 000 kg CO_2_e.

**Clinical implications** Psychiatrists need to review their prescribing practice of long-acting injections to reduce their impact on the National Health Service financial budget and the environment.

The National Health Service (NHS) has committed to meeting the targets of the Climate Change Act 2008, which entails reducing its carbon footprint by 80% by 2050. As it stands, the carbon footprint for NHS England is around 25 million of CO_2_e (carbon dioxide equivalent), of which mental health services account for about 6%.^1^ Therefore, between now and 2050 the necessary carbon reductions are in the order of 20 million tonnes of CO_2_e, of which about 1.2 million tonnes will need to come from mental health services (assuming reductions occur proportionally).^2^ Meeting these carbon reduction targets will require a transformation in the way mental healthcare is delivered,^3^ as the main component of the carbon footprint of healthcare is not its buildings or energy use (only 17%), but factors relating to clinical practice. The single largest component of this for mental healthcare is pharmaceuticals.^2^

One way of reducing greenhouse gas emissions without compromising the quality of care is to eliminate inefficiencies in service provision.^4^ In this paper we explore one potential area for improvement – the overprescribing of long-acting injections for the treatment of mental illness. More specifically, we assess the prescribing patterns of injections of flupentixol decanoate for the treatment of schizophrenia and identify areas of wastage.

We chose flupentixol because it is the most commonly administered long-acting injection in the UK at 15 000 items per year.^5^ Furthermore, there is evidence that it is being prescribed at higher doses and more frequently than studies suggest is beneficial. The average prescription in the UK is 60 mg every 2 weeks^6^ and the licensed dose limit is 400 mg per week, but a Cochrane review (based on two small studies) found no evidence for clinical improvement from doses higher than 50 mg every 4 weeks.^7^ Admittedly, randomised controlled trials are poorly representative of naturalistic samples, but a more recent study systematically reviewed eight studies, again with small numbers and significant heterogeneity, and came to the same conclusions about dose.^8^ Despite the small evidence base, evidence suggests there is a maximum clinically effective dose that is considerably less than the average prescribed dose.^8^

We used data from Oxford Health NHS Foundation Trust and extrapolated to the national level to quantify the potential economic and environmental impact to the NHS of prescribing flupentixol decanoate at higher doses and higher frequency than is clinically beneficial.

## Method

We collated the prescription details of all patients receiving flupentixol decanoate at Oxford Health NHS Foundation trust in December 2013. The month was chosen at random and, given the long-term nature of these prescriptions, there is no reason to expect it to differ from other months.

Included in the data was information about the medication prescribed, the materials used to administer it (syringe, needle, glass vial and packaging), the number of appointments and travel to and from the appointment for both patients and staff. Travel data were not from the same patients, but were obtained from a survey conducted independently by the Trust during the same period that included 100 rural and 100 urban patients (details available from the authors on request). No information was available on cleaning materials and wasted medication so we have assumed that neither makes a meaningful contribution to overall resource use. The financial cost of heating and lighting the clinic rooms were also excluded due to lack of data.

Conversion factors used to estimate the carbon footprint associated with the materials are presented in Table 1 and come from either the Department for Environment, Food & Rural Affairs or the NHS Sustainable Development Unit. In order not to overestimate the impact, we assumed the use of recycled materials, although this is probably a conservative assumption. We assumed that an injection was administered using a standard NHS 5 mL, 21G-VanishPoint intramuscular needle and syringe and a 1 mL vial. For transport, we assumed small to average-size cars. For the financial costs of medication, the cheapest available estimates were used as derived from the *British National Formulary 67* (BNF; www.bnf.org). The costs of needles and syringes were from the NHS supply chain data (www.supplychain.nhs.uk) and the cost of appointments from national databases.^9^ The administration of an injection was assumed to require a 15-minute out-patient appointment with a band 5 nurse and the costs included all overheads.

**Table 1:** Data used in analysis, obtained from other sources

Data	Source	Amount/unit
Carbon footprint of travel	Trust travel survey^a^	1.87 kg CO_2_e/appt

Cost of travel	Trust travel survey^a^	£2.12/appt

Cost of needle and syringe	NHS supply chain 2013^9^	£0.24/appt

Carbon footprint of needle and syringe	DEFRA conversion factors (kg CO_2_e/tonne)^13^	0.0162 kg CO_2_e/appt^b^

Carbon footprint of energy use in appointment	Sustainable Development Unit 2013^2^	13 kg CO_2_e/appt

Cost of appointment	Unit Costs of Health and Social Care 2013^9^	£19/15 min appt with band 5 nurse^c^

Carbon footprint of medications per £1 spent	Sustainable Development Unit 2013^2^	0.43 kg CO_2_e/£

Cost of FD injection	*British National Formulary* 2013	0.0625£/mg

National cost of FD injection prescriptions	NHS Business Services Authority 2009^5^	£210 000/year in England

National number of FD injection prescriptions	NHS Business Services Authority 2009^5^	15 000 prescriptions/year in England

Maximum effective dose of FD	Cochrane review^7^	50 mg/4 weeks

appt, appointment; DEFRA, Department for Environment, Food & Rural Affairs; FD, flupentixol decanoate; kg CO_2_e, kilograms carbon dioxide equivalent; NHS, National Health Service.

a. Travel survey at Oxford Health NHS Foundation Trust, December 2013. Data available from the authors on request.

b. Calculated as a sum of needle production factor (1222.0), syringe production factor (2.138.0), paper packaging production factor (954.5), glass vial production factor (508.0) and waste factor for each material if recycled (21).

c. Including all overheads, administrative support, buildings, etc.

We made two assumptions in calculating the costs and carbon footprints. First, if flupentixol decanoate was prescribed, it was assumed to be administered and second, the national average interval was assumed to be the same as that found for Oxford Health NHS Foundation Trust, and not the national average of 2 weeks. The latter again entails a conservative estimate of the economic and environmental costs. For the national cost of flupentixol, we used actual national data; all other costs were extrapolated from Oxford data.

We calculated the economic and environmental savings that would occur if all patients were given flupentixol decanoate injections according to best practice, that is 50 mg every 4 weeks. We did this by identifying the resources used to administer one injection; this involved measuring all resources used and then attaching a financial and environmental cost to the resources, as explained earlier. Annual costs per organisation were calculated presuming that each organisation had the same number of patients on long-term flupentixol decanoate injection, as was the case for Oxford Health NHS Foundation Trust. National costs were calculated based on national data that 15 000 prescriptions of flupentixol decanoate were issued at a cost of £210 000 per year.^5^

## Results

### Organisation-level analysis

During December 2013, 28 patients attended 59 appointments for flupentixol decanoate injection at Oxford Health NHS Foundation Trust. The average interval was found to be 2.2 weeks, which is longer than the national average of 2 weeks. The average dose was lower than the national average: 46 mg per week (101 mg per injection) *v*. 60 mg per week.^6^ There was considerable variation in both prescribing interval and dose for patients. Prescribing intervals ranged from 1 to 4 weeks and the dose ranged from 40 mg to 300 mg per week. The annual cost of providing flupentixol decanoate to 28 patients at the Trust was £18 012 and its annual carbon footprint was 11 519 kg CO_2_e (Table 2).

**Table 2:** Economic and environmental costs of flupentixol decanoate per year for Oxford Health NHS Foundation Trust

Resource	Financial cost £	Financial cost burden %	Carbon footprint kg CO_2_e	Carbon cost burden %
Medication	3876	22	1668	14

Needle and syringe	156	1	11	<1

Appointment	12 576	70	8604	75

Travel	1404	8	1236	11

Total	18 012	100	11 519	100

### Extrapolation to the national level

In estimating national costs, all figures remained the same and were scaled up to the national level except for the cost of medication, which was given as £210 000 spent on flupentixol decanoate injection per year for 15 000 prescription items.^5^ This is costing the NHS in England around £530 000 per year. Over 50% of these costs are due to the cost of staff in the appointment at £285 000, followed by medication at £210 000. The carbon footprint of this service amounts to over 314 000 kg CO_2_e across England, which is mostly attributable to the energy required for the appointment (195 000 kg CO_2_e) and medication (90 300 kg CO_2_e). The economic and environmental costs of materials used for the injection are minimal. The costs of travel are not large but remain noteworthy at around £31 800 and 28 000 kg CO_2_e per year for England (Table 3).

**Table 3:** Projected economic and environmental costs of flupentixol decanoate per year for England

Resource	Financial cost £	Financial cost burden %	Carbon footprint kg CO_2_e	Carbon cost burden %
Medication	210 000	40	90 300	29

Needle and syringe	3624	1	244	<1

Appointment	285 000	54	195 000	62

Travel	31 800	6	28 050	9

Total	530 424	100	313 594	100

Analysis of trust-level and national-level data reveals that the dominant financial cost and carbon burden are associated with the appointment. Medication is ranked in second place, whereas medical consumables and travel are less significant costs and burdens. At a national level, medication contributes a larger proportion; this is because the cheapest BNF cost was used when analysing the Trust data.

### Potential savings associated with evidence-based administration

Considerable environmental savings could be achieved across England by changing prescribing behaviour to adhere to best practice.^6,7^ Analysis suggests that around 166 000 kg CO_2_e could be saved, most of which is attributable to the carbon footprint of the energy used in the appointment (88 000 kg CO_2_e) and the potential over-prescribing of medication (66 000 kg CO_2_e) (Table 4). This equates to a saving of 168 kg CO_2_e per patient per year.

**Table 4:** Projected reductions in economic and environmental costs that could be achieved by increasing interval of injection to maximum 4 weeks and reducing dose to maximal effective dose

	Potential financial savings, £/year		Potential carbon footprint savings, kg CO_2_e/year	
Resource	For each patient	Nationally	For each patient	For England
Medication	22	152 935	10	65 762

Needle and syringe	3	1631	0	110

Appointment	202	128 250	138	87 750

Travel	23	14 310	20	12 623

Total	250	297 126	168	166 245

Considerable financial savings could also be achieved by changing prescribing practices. The calculations suggest that around £297 000 could be saved across England by improving prescribing behaviour, which equates to £250 per patient per year (Table 4).

## Discussion

This paper demonstrates that appropriate dosing of flupentixol decanoate would have economic and environmental benefits for the NHS. If all prescriptions across England were given at the maximum 4-week interval and at no more than the evidence-based maximal effective dose, around £300 000 could theoretically be saved. This change in prescribing practice would also lead to saving around 166 000 kg CO_2_e across England per year. This potential overprescription of flupentixol decanoate injection has the effect of increasing a patient's annual carbon footprint by about 170 kg CO_2_e over and above that necessary (Table 4), which is the equivalent of a 2% increase of the average carbon footprint per person in the UK (from 7.9 to 8.1 tonnes CO_2_).^10^ These savings also mean that, for this particular service, they would also go a long way to meeting the Climate Change Act targets of an 80% reduction in carbon footprint.

This analysis, although only based on 28 patients, could be generalised to other settings. Rural settings would have an increased proportion of costs associated with travel, whereas prescribing practices are likely to vary across regions, as are team and individual management plans for patients on flupentixol decanoate. Furthermore, some mental health trusts have renewable energy sources on site that might considerably reduce the carbon footprint associated with building energy use.

Some of these savings may not materialise in reality, as staff time and resources are likely to be used for other patients. Furthermore, the energy used to heat and light the building is unlikely to reduce much by reducing appointment frequency, as the clinical facility is likely to have other uses. It is, however, an important principle to reduce use of unnecessary resources as it can enable potential savings to be used more effectively elsewhere.^11^ This process creates a higher-value healthcare system where resources such as funding, carbon and staff time are released from some parts of the system to develop new services or support struggling services.

There are two main issues that lead to unnecessary financial expenditure and emissions of carbon dioxide. The first is the prescribing of medication at doses higher than evidence suggests is beneficial and the second is the administration of the injection at shorter intervals than is necessary.^12^ Current trends of overprescribing may be attributable to prescribing habits, personal experience or a result of the historical use of higher doses. Overprescribing may also be due to a notable clinical benefit at higher doses in some individuals.

An important finding here is that appointments appear to be a major component of the carbon footprint for mental health services. If these could be reduced, without compromising care, major savings could occur. In this study, increased appointments are driven by the shorter intervals between doses. Reasons for this might include convenience, efficacy and the view that some patients deteriorate in the days before the next dose is due, although this is not supported by evidence.^6^ Patients are often initially prescribed injections at 2-weekly intervals, but perhaps this interval is not reviewed at subsequent appointments leading to unnecessary use of appointments and environmental resources. However, some patients are administered injections every 2 weeks because it is clinically necessary to maintain a 2-weekly clinical review and it is considered logical that the injections should be administered at the same time. If these patients are also prescribed within the evidence-based dose, then the savings associated with medication cannot be included in this analysis; neither can the savings attributed to the travel. In such cases, the potential savings associated with increasing the interval of the injection are due to the increased length of time needed during each appointment to administer the injection and the material costs of the needle and syringe. In this particular instance, based on reviewing the percentage burden from each component in the analysis, the savings associated with the increased time for each appointment are likely to be important but the material use will not be (less than 1% of total burden). Thus, if the appointment did not increase in its duration, then there would be no incentive to increase the interval of the injection to 4 weeks.

Current prescribing practices can have a detrimental impact across the three components of sustainability: environmental, economic and social. The environmental and economic costs have been outlined, however, the social costs may also be substantial, including increased frequency of painful injections, time spent by the patient attending unnecessary appointments, and an increased risk of extrapyramidal side-effects.^6^ The wider social cost of these side-effects is also likely to be negative and may include reduced socialisation, reduced employment and larger healthcare costs, which in turn will increase the carbon burden and financial cost associated.

More evidence is needed to more clearly establish the maximally effective dose of flupentixol decanoate as the implications of changing prescribing behaviours can be substantial for the patients affected. However, if current practice does not follow the current available evidence, will additional economic and environmental savings make any impact on doctors' behaviours? Perhaps a culture change at all levels is required to recognise the importance of reducing wasteful practice and to develop a sense of stewardship over the use of clinical resources.
